# Peri-Implantitis Diagnosis and Prognosis Using Biomarkers in Peri-Implant Crevicular Fluid: A Narrative Review

**DOI:** 10.3390/diagnostics9040214

**Published:** 2019-12-07

**Authors:** Hatem Alassy, Praveen Parachuru, Larry Wolff

**Affiliations:** Division of Periodontology, Department of Developmental and Surgical Sciences, School of Dentistry, University of Minnesota, Minneapolis, MN 55455, USA; parac069@umn.edu (P.P.); wolff001@umn.edu (L.W.)

**Keywords:** peri-implantitis, biomarkers, peri-implant crevicular fluid, peri-implant sulcular fluid, mucositis, implant disease

## Abstract

Dental implant diseases, peri-implantitis (PI) and peri-implant mucositis (PIM), have shown wide prevalence in recent studies. Despite the prevalence, diagnosing peri-implant disease (PID) remains challenging as common diagnostic methods of periodontal probing and radiographs may be inaccurate. These methods only document pre-existing destruction rather than current disease activity. Furthermore, there is no current model to predict the progression of PID. Though a predictive model is lacking, biomarkers may offer some potential. Biomarkers are commonly used in medicine to objectively determine disease state, or responses to a therapeutic intervention. Gingival crevicular fluid (GCF) biomarkers have moderate diagnostic validity in periodontitis. Biomarkers in peri-implant crevicular fluid (PICF) also show promising results in regard to their diagnostic and prognostic value. The aim of this review is to summarize the current knowledge of PICF biomarkers in the diagnosis of PID and evaluate their validity to predict disease progression. This review found that PICF studies utilize different methods of sampling and interpretation with varying validity (sensitivity and specificity). A number of promising diagnostic techniques were identified. Commercially available chair-side tests for MMP-8 to diagnose periodontal disease and PID activity are now available. Future directions include proteomics and metabolomics for accurate, site-specific diagnosis and prediction of PID progression. Although more research is needed, this review concludes that the assessment of proinflammatory cytokines (IL-1β, TNFα, MMP-8) in the PICF may be of value to diagnose PI and PIM but current research remains insufficient to indicate whether biomarkers predict peri-implant disease progression.

## 1. Introduction

Teeth may be lost for several reasons and surgically placed dental implants are one replacement option [1]. There has been a significant increase in the prevalence of dental implants among adults in the United States. From 1999 to 2016, an increase of 14% in implant prevalence per year was noted. Six percent of adults in the U.S. had benefited from dental implants by 2016. Dental implant prevalence among U.S. adults could be 17% by 2026 if the trend continues at the current pace [2].

As implants are becoming more common, associated disease prevalence shows a positive correlation. Peri-implant mucositis (PIM) is defined as an inflammatory lesion of the soft tissues surrounding an endosseous implant without loss of supporting bone or continuing marginal bone loss [3]. On the other hand, peri-implantitis (PI) is a pathological condition occurring in tissues around dental implants, characterized by inflammation in the peri-implant connective tissue and progressive loss of supporting bone [4]. According to a meta-analysis and systematic review, the estimated prevalence of peri-implantitis is 22% and peri-implant mucositis is 43% [5]. The absence of signs of clinical inflammation is considered peri-implant health [6].

There is a wide heterogeneity in defining peri-implantitis. Clinicians seek to differentiate how much radiographic bone loss would be indicative of disease, as opposed to the expected post-placement bone remodeling. The radiographic criterion for peri-implantitis is defined as bone loss of ≥3 mm according to the 2017 World Workshop on the Classification of Periodontal and Peri-implant Diseases and Conditions [7]. In the absence of initial radiographs and probing depths, radiographic evidence of bone loss of ≥3 mm and/or probing depths ≥6 mm in conjunction with profuse bleeding fits the definition for PI.

Progression of PI was found to have an annual rate of bone loss of about 0.4 mm but in a non-linear and accelerating pattern [8]. Peri-implantitis often appears within the first few years after the implant is in function [1]. Plaque/biofilm is a principal etiological factor; except for some unusual factors such as implant fractures and iatrogenic errors. It has been shown that there is an increased risk of developing PI in patients who have a history of severe periodontitis, poor plaque control, and no regular maintenance care after implant therapy [7].

Destructive periodontal diseases are the result of environmental, host, and bacterial factors [9].

Similarly, PI exhibits a chronic inflammatory response to the bacterial biofilm on the tooth/implant surface [10]. PIM precedes PI and the progression of PI appears to be faster than periodontitis around natural teeth [4]. PIM is primarily caused by a disruption of the host–microbe homeostasis at the implant–mucosa interface and is a reversible condition [3]. There is no consensus on which surgical intervention is most reliable in controlling peri-implantitis [11].

No one specific or unique bacteria has been identified in patients with peri-implant disease (PID). When compared with healthy implant sites, PI was associated with higher counts of 19 bacterial species, including *Porphyromonas gingivalis* and *Tannerella forsythia* [4]. When compared to periodontitis in natural teeth, PI was more frequently linked with opportunistic pathogens of bacterial, fungal and viral origins which points to a heterogenous infection [4]. Some individuals are believed to be more susceptible to peri-implantitis. Current evidence indicates a potential influence of various gene polymorphisms in the pathogenesis of peri-implantitis; however, prospective clinical studies with sufficient sample size are currently lacking [4]. Gram-negative bacteria are the most important bacteria frequently isolated from the periodontal pockets of natural teeth, such as: *Aggregatibacter actinomycetemcomitans, Eikenella corrodens, Fusobacterium nucleatum, Prevotella intermedia, Porphyromonas gingivalis and Tannerella forsythia* [12]. However, a recent systematic review pointed out the importance of new pathogens, such as Desulfobulbus spp., Filifactor alocis and TM7 spp., in periodontal disease [12]. Notably though, periodontal disease around natural teeth is probably not caused by the presence of specific bacteria, but by changes in the levels of the population of the species in the oral microbiome.

The traditional clinical method to assess implant health includes a periodontal probe to measure the pocket depths and to observe bleeding upon probing. Unfortunately, this simple tool has limitations. The absence of a periodontal ligament around implants and the prosthetic design may make assessment of pocket probing depth measurements difficult to perform and interpret. Additionally, the implant mucosal seal may have less resistance to probing compared to natural teeth. This may lead to mechanically induced bleeding when probing around healthy implants. However, the healing of the epithelial attachment seems to be complete five days after clinical probing, hence, does not seem to jeopardize the longevity of implants according to an animal study [13]. Radiographs should be standardized and compared to reference radiographs taken at the time the implant was placed in function. Furthermore, there is no practical model to predict the progression of PI [1]. Predicting disease progression is an essential component to form a prognosis. Treatment protocols cannot be easily compared without a valid prognosis. Non-surgical therapy of PI is often ineffective, and the treatment of choice is a surgical approach [11]. Surgical techniques may include open flap debridement with removal of the inflammatory tissue and mechanical and chemical decontamination of the exposed implant surface. Recontouring of the bony architecture and smoothing of the implant surface may improve infection control. Regenerative procedures using a membrane and bone graft substitutes attempting to partially fill the bony defects caused by peri-implantitis can be successful [14]. Therapy of peri-implantitis followed by regular supportive care resulted in favorable clinical improvements and stable peri-implant bone levels in the majority of patients according to a systematic review [15].

Early diagnosis of PID and its rate of progression are a great challenge. Assessment of biomarkers may aid in early detection of PI. Biomarkers may assist both in staging and grading of periodontitis in the case definition system of periodontitis [16]. Peri-implant crevicular fluid (PICF), also described as peri-implant sulcular fluid (PISF), may contain biomarkers to diagnose and predict future disease which aids in choosing a specific treatment protocol. A biomarker is a parameter that is objectively measured and evaluated as an indicator of normal biological, pathogenic processes, or responses to a therapeutic intervention [17]. Molecules in the gingival crevicular fluid (GCF) collected from natural teeth have been extensively studied. Substances such as lactate dehydrogenase and myeloperoxidase have been investigated to determine if they could be used as markers for periodontal pathology and in the success of treatment modalities [18]. Another approach was described in a recent report which found that measuring glycosylated hemoglobin in gingival crevicular *blood* was successfully used to screen for diabetes control in a dental office setting [19]. The aim of this review is to summarize the current application of peri-implant crevicular fluid (PICF) substances as biomarkers for peri-implantitis.

A literature search was performed in the Pub-Med database of the US National Library of Medicine, for articles published up to October 2019 using Medical Subject Heading search terms + free text terms and in different combinations. To be included in the review, studies had to (i) be written in the English language, (ii) be published in an international peer-reviewed journal, and (iii) be on humans, while animal or in-vitro studies were supplemented by an additional search to find additional relevant supporting data. The search yields were transferred to Endnote™, version 9 (Clarivate Analytics, Boston, MA, USA). Citation tracking was completed for all identified studies included in the refined library. No restriction was placed on the year of publication for the included reports. Keywords and abbreviations used in this narrative review are shown in Table 1. Most of the PI biomarker studies summarized in this review focused on pro-inflammatory cytokines, enzymes and bone metabolism proteins. These were the main categories of biomarkers found in our literature search. Other related substances which were found in this search can be found in Section 2.4.1.

## 2. Discussion

### 2.1. Gingival Crevicular Fluid and Peri-Implant Crevicular Fluid in Health and Disease

GCF is a physiological fluid and an inflammatory exudate originating from the gingival plexus of blood vessels in the gingival corium, subjacent to the epithelium lining of the dentogingival space. GCF flows through the external basement membrane and the junctional epithelium to reach the gingival crevice. The composition of GCF can potentially be used to detect subclinical alterations in tissue metabolism, inflammatory-cell recruitment and connective tissue remodeling [20]. Cytokines and enzymes located in the gingival tissues may lead to the degradation of connective tissue collagen and alveolar bone. These are host response factors from local host tissue reacting to the plaque biofilm. In the presence of disease, the volumes of GCF and PICF were similarly higher than in healthy sites, and GCF flow increases with an increase in the severity of gingival inflammation [21]. Significant positive correlations were noted between the concentrations of cytokines in PICF versus their levels in GCF around natural teeth [22]. Another study compared the cytokine and bacterial levels from around implants versus teeth within the same individual [23]. There were many similarities but, also some differences in levels of IL-1β and soluble receptor activator nuclear factor kappa-B ligand (sRANKL) and bacterial species between peri-implant and periodontal sites in the same individuals, suggesting similar pathogenic mechanisms. Investigators compared crevicular fluid from diseased teeth and implants, and tested collagenase activity and collagenolytic matrix metalloproteinase (MMP) levels. Results indicated that peri-implantitis PICF contained higher active MMP-8 levels than GCF from similar deep chronic periodontitis sites of natural teeth [24]. A 10-year retrospective investigation comparing crevicular fluid biomarkers from implants and teeth concluded that increased levels of MMP-8 and IL-1β in PICF or GCF may be associated with inflammation around implants and teeth while lower levels of MMP-1/TIMP-1 may be an indicator of disease progression around implants [25].

### 2.2. PICF Disease Mediators (Cytokines)

The gingival sulcus forms a unique ecological niche for microbial colonization. As the salivary film transitions at the crown to the gingival sulcus, its composition changes and the proportion of serum proteins increases due to the proximity with the crevicular fluid. The microbiota of the dental plaque biofilm drives the inflammatory process. The microbial biofilm in the gingival sulcus elicits inflammation in the surrounding connective tissue [26]. The imbalance between the bacterial challenge and host response at the soft tissue–implant interface triggers an inflammatory process [27]. Cytokines, such as Tumor necrosis factor alpha (TNFα), Interleukin-1-beta (IL-1β) and Interleukin-6 (IL-6), are released from cells of the gingival epithelium, dendritic cells, connective tissue fibroblasts, macrophages and neutrophils. In addition, a number of enzymes, such as matrix metalloproteinases, are produced by neutrophils, fibroblasts and osteoclasts, leading to the degradation of connective tissue collagen and alveolar bone [20]. More than 90 different molecular components in GCF have been evaluated for potential periodontal disease diagnosis associated with the natural dentition [28]. To date, significantly fewer PICF components have been analyzed around implants.

PI and periodontitis lesions exhibit critical histopathologic differences, which contribute to the understanding of dissimilarities in onset and progression between the two diseases [29]. Histologically, PI lesions extend apical to the junctional epithelium and contain large numbers and densities of plasma cells, macrophages and neutrophils. PI lesions are larger than those found at PIM sites [7]. In contrast to periodontitis, PI lesions are more than twice the size and contain a significantly larger area, numbers, and densities of CD138−, CD68−, and MPO-positive cells. Furthermore, larger densities of vascular structures are seen in the connective tissue area lateral to the infiltrated connective tissue than within the infiltrate in PI lesions [29].

### 2.3. PICF Research in Peri-Implantitis: Methods of Sampling and Analysis

A variety of methods have been used to sample and analyze components of PICF targeting the diagnosis of PI. The most common method used to collect PICF has been paper strips inserted into the crevice for typically 30 s. PICF is then absorbed onto the strips. After elution from the strips into a buffer or diluent, the fluid is evaluated utilizing biomarker-specific assays. Studies have also used paper cones, membranes and microcapillary pipettes to collect PICF.

The PICF volume is dependent upon the level of inflammation and pocket depth. The quantity of components collected in a deep and inflamed pocket would in all likelihood be higher than in healthy sulci. The concentration versus the quantity of the collected PICF components may offer more value in the search of valid biomarkers; however, this may be controversial [30]. Enzyme-linked immunosorbent assay (ELISA), flow cytometry, Luminex and Spectrophotometry were the most utilized assays in PICF research. Typically, studies used ELISA to analyze PICF and evaluated one to two cytokines [31].

### 2.4. What Studies Evaluated

#### 2.4.1. PICF Molecules Investigated

Most PI biomarker studies analyzed the correlation of enzymes and cytokines between healthy and diseased implants. Such components include proinflammatory cytokines such as TNFα, IL-1β, IL-6, IL-12, IL-17 and anti-inflammatory cytokines such as IL-4 and IL-10. Other cytokines analyzed were interleukin-1 receptor antagonist (IL-1ra), Granulocyte-macrophage colony-stimulating factor (GM-CSF) and salivary IL-1β. Some studies investigated chemokines such as IL-8 and macrophage inflammatory protein-1α (MIP-1α). Other reported investigations analyzed bone metabolism markers such as Receptor activator nuclear factor kappa-B ligand (RANKL), Receptor activator nuclear factor κ B (RANK), Osteoprotegerin (OPG) and Osteocalcin. Other trials investigated enzymes such as matrix metalloproteinases (MMP1, MMP8, MMP9, MMP13), tissue inhibitor of metalloproteinases 1&2 (TIMP), Myeloperoxidase (MPO), Elastase, tissue plasminogen activator (tPA), tartrate-resistant acid phosphatase (TRAP), cathepsin K (CatK). Some authors reported on other PICF components such as plasminogen activator inhibitor 2 (PAI-2), vascular endothelial growth factor (VEGF), prostaglandin E2, KLIKK-protease genes and miropin.

#### 2.4.2. Proinflammatory Cytokines

Most investigations including systematic reviews and meta-analyses focused on the assessment of proinflammatory cytokines IL-1β and TNFα levels, demonstrating that PI sites were associated with a significant increase in their levels compared to healthy implants. IL-1β and TNFα are the two most important cytokines in osteoclast formation and bone resorption. IL-1β is mainly produced in macrophages and regulates the degradation of extracellular matrix components of the plasminogen system and collagenase activity in inflammation and wound healing. It has been shown that inhibition of IL-1β reduces tissue breakdown and the progression of tissue inflammation [32]. TNFα induces fibroblast apoptosis and reduction of the repair capacity of the peri-implant tissue [33]. Statistical differences were revealed when IL-1β and TNFα levels were compared between healthy implant sites and PID sites. No statistical differences could be detected between PIM and PI [33]. There is limited evidence presented in published literature that other proinflammatory cytokines (IL-6 and IL-17) have higher levels in PI compared to crevicular fluid associated with healthy implants [34,35]. Contrary to what was found with IL-1β, IL-6 increases significantly between PIM and PI. IL-6 links innate and acquired immune responses, in which it induces differentiation of activated B cells into antibody-producing cells as well as naïve CD4+ T cells [36]. Studies of experimental PIM demonstrated that TNFα and TGF-β2 levels did not change during an experimental PIM period. IL-1β yielded a significant increase after 3 weeks of cessation of oral hygiene measures and was reversed to pre-experimental levels 69 days after oral hygiene measures were reinstated [37].

#### 2.4.3. Anti-Inflammatory Cytokines

IL-10 in PICF has been shown to be negatively correlated with peri-implant disease [35,38]. IL-4, -8 and -12 showed no differences between health and PID [31]. Chemokines IL-8 and Macrophage inflammatory protein-1α (MIP-1α) were higher in diseased sites [39,40]. However, to our knowledge, these findings were not corroborated by other studies.

#### 2.4.4. Bone Loss Markers

Since RANKL and OPG are key factors regulating bone metabolism, it is likely they are involved in alveolar bone destruction in PI [41]. However, the majority of studies failed to identify any significant differences in the levels of bone metabolism markers between healthy and PI sites [42]. Soluble RANKL and OPG levels were evaluated in 84 samples of PICF from implants showing different peri-implant tissue clinical conditions without demonstrating any correlation between these levels and the studied clinical outcomes [43].

However, one study demonstrated the presence of OPG in 79% of the PICF samples and showed a significant positive correlation between BOP, while sRANKL was only detected in 12% of PICF samples and did not show any positive correlation with clinical inflammation [41]. Another study compared the levels of C-telopeptide pyridinoline cross linkage of type I collagen (ICTP), sRANKL and OPG in PICF [44]. The results demonstrated an increase in total amount of ICTP and OPG in the PI group when compared to the healthy group. However, sRANKL was not significantly different between the healthy implant and diseased implant groups. Alternatively, another report showed significantly higher levels of sRANKL, OPG and RANK in PI sites compared to healthy implant sites [45]. However, the OPG/sRANKL ratio demonstrated no significant difference between the healthy and diseased implant groups. Osteocalcin, osteopontin and osteonectin proteins are related to bone remodeling. There were no significant differences in PICF osteocalcin, osteopontin and osteonectin total amounts between healthy controls, peri-implant mucositis and peri-implantitis groups in one recent study [46].

An interventional trial assessed the effects of mechanical anti-infective therapy on the levels of TNFα and OPG/RANKL ratio in healthy implant and PI sites [47]. The results demonstrated significantly higher levels of TNFα in PI sites when compared to healthy sites. The OPG/RANKL ratio was shown to be low in healthy sites compared to PI sites. After mechanical anti-infective therapy, TNFα levels were significantly reduced in treated diseased sites and reached the same level as in healthy control sites at 3 months post therapy.

#### 2.4.5. Enzymes

Certain enzymes in PICF, such as cathepsin K and MMPs, were heavily investigated. Cathepsin-K (CatK) is a cysteine protease that is highly expressed by osteoclasts. Its main function is hydrolyzing the extracellular bone matrix proteins. CatK was shown to be elevated in GCF from chronic periodontitis sites compared to healthy sites. CatK is a known marker of bone turnover due to its key role in remodeling and cartilage breakdown in bone by hydrolyzing extracellular bone matrix proteins. CatK is highly and quite selectively expressed in active, resorbing osteoclasts [48]. This suggests its role in the pathogenesis of PI. CatK levels were evaluated in PICF to assess the levels of CatK in healthy implants and PI in order to correlate these findings with clinical parameters. Some investigations showed a positive correlation between clinical parameters of PI and levels of CatK [49,50] while others concluded that CatK showed no differences between the healthy and diseased implant groups [39].

MMP upregulation has been associated with irreversible peri-implant connective tissue destruction [42]. One suggested reason for MMP upregulation is polymorphism in the promoter region of MMP-8 which explains varied responses between different individuals with the same disease category [36]. During the initiation and course of inflammatory responses in PI, proinflammatory mediators including MMP-8 are up-regulated in affected tissues and present in PICF [51]. IL-1β and TNFα induce the synthesis and secretion of MMP-8, which in turn, cleaves the triple helix of collagen and collectively degrade the extracellular matrix [36]. Similar to what was found in periodontitis, there is moderate evidence in the literature showing high MMP-8 levels in PI compared to healthy implant sites [25,36,52,53,54]. MMP-8 is a promising biomarker as an early signal of peri-implant inflammation [52].

Another investigation reported a positive correlation between MMP-8, PI, and BOP in both GCF and PICF [25]. The authors concluded that increased levels of MMP-8 and IL-1β in PICF or GCF may be associated with inflammation around teeth and implants while lower levels of MMP-1/TIMP-1 may be an indicator of disease progression around implants. Other clinical trials demonstrated that MMP-8, MMP-9 and MMP-13 (also known as Collagenase-2, Gelatinase B and collagenase-3, respectively) in PICF were associated with more bone loss around diseased implants, indicating that MMP-8 could be a promising biomarker for peri-implant osteolysis [53,55]. However, a different trial concluded that MMP-8 did not reveal a meaningful difference to differentiate PI from healthy implants [27]. IL-1β, VEGF, and TIMP-2, *T. denticola* and *Prevotella intermedia* showed diagnostic validation for PI in this study. IL-1β demonstrated the most significant ability for the prediction of PI disease status (sensitivity: 0.73, specificity: 0.73, odds ratio: 7.71). The authors concluded that a combination of the above markers and microbial profiles may offer site-specific diagnosis of PID due to the increased sensitivity and specificity compared to individual biomarkers [27]. A summary of the commonly investigated and promising peri-implant crevicular fluid biomarkers is shown in Table 2.

### 2.5. PICF Biomarkers: Chair-Side Diagnostic Tests, Limitations

#### 2.5.1. Chair-Side Diagnostic Tests

If a definitive diagnosis for PI or PIM can be made using a test with high validity, then a reasonable question would be “is the test feasible and can the test can predict progressive PI?”. Clinicians seek an easy, accurate, inexpensive and time effective test. Most diagnostic tests in dentistry are performed in a clinical setting at the dental chair. A chair-side test for the diagnosis of PIM would also be valuable if it can predict the risk of disease progression at an implant site.

A recent report presented a diagnostic chewing-gum test which could detect elevated levels of MMP-8, which was diagnostic of PI in individuals [56]. The patient would taste bitterness if MMP-8 was elevated in the oral fluids. To our knowledge, a randomized controlled clinical trial has not been conducted to determine if the chewing gum test is diagnostic of PI [56]. Elevated levels of MMP-8 in PICF are associated with peri-implant inflammation while low MMP-8 levels (<20 ng/mL) indicate healthy peri-implant tissues [57]. Pathologically elevated levels of MMP-8 (>20 ng/mL) can be detected by a quantitative MMP-8 chair-side device, ImplantSafe^®^ [57]. This is a chair-side and point-of-care test for the detection of MMP-8 in oral fluids and is commercially available. The ImplantSafe^®^ device may be useful in differentiating active from inactive periodontal and peri-implant sites easily, quickly and inexpensively with high sensitivity and specificity (sensitivity of 90% and a specificity of 70%–85%) [57]. Chair-side diagnostic tests may assist to determine implant therapy success. Furthermore, this device is said to detect subclinical, developing periodontitis and peri-implantitis before the appearance of clinical and radiographic signs [58]. This ability to predict future disease is a significant advantage in preventing irreversible damage to dental implants. The quantitative results of this chair-side test may be beneficial in assessing the rate of disease progression; however, future prospective studies would be important to ensure the validity of this test in predicting PI.

#### 2.5.2. Limitations of Studies

The conflicting results of the above studies may be due to differences in study design, material and methods utilized, such as sample collection, processing and assay sensitivity. Meta-analyses in PICF biomarkers are scarce due to heterogeneity between studies in PI diagnosis criteria. The 2017 World Workshop hopes to utilize their PI definition criteria in future clinical and research settings [1]. If some studies set their PI definition as radiographic bone loss of 3 mm while other studies had a 2 mm cut off, then the PICF results of these investigations may not be accurately combined and analyzed. There is a wide range of different definitions regarding peri-implant mucositis and peri-implantitis that were employed in the included investigations. The definition of peri-implantitis varied over time, mainly from more permissive to stricter inclusion criteria. In light of the new definition of peri-implantitis by the 2017 World Workshop [1,7], some of the reviewed studies may have included cases of peri-implant mucositis in the group of peri-implantitis. Such misdiagnosis of peri-implantitis and inclusion of cases of peri-implant mucositis may affect the results of studies on PICF.

The majority of PICF studies assessed only a few cytokines, enzymes or pathogens to correlate them with peri-implant diseases. Most studies lacked data on sensitivities and specificities to biomarkers in PICF; hence, the probability of false positive or false negative results could not be calculated. PICF biomarkers analyzed individually have shown mixed results or low sensitivity and high specificity values, which may weaken the biomarker’s disease predictive value. On the other hand, biomarkers of periodontal disease progression in GCF alone (MMP-8, MMP-9, Osteoprotegerin, C-reactive Protein and IL-1β) provided low sensitivity and high specificity values of 23% and 95%, respectively [59]. It is noteworthy that, combined with plaque pathogens, GCF biomarkers demonstrated the highest positive and negative predictive values of 73% and 70%, respectively [59]. Similarly, selected PICF-derived biomarkers of periodontal tissue inflammation, matrix degradation/regulation, and alveolar bone turnover/resorptive molecules combined with a site-specific microbial profile may be used to diagnose peri-implant diseases [27]. Another multi-biomarker approach presented a 3-biomarker model (IL-17, IL-1ra and VEGF) distinguishing healthy implant PICF from PID subjects with high validity (AUC: 0.90) [35].

Some reports failed to discuss important data, such as the general periodontal health in their subjects, smoking habits, systemic confounders, and other related criteria which may influence the levels of cytokines in PICF. Smoking may be an important confounder because it may affect the PICF volume and cytokine levels. Only a few studies excluded smokers. Other potential confounders may include a history of periodontitis and gingival phenotype. A retrospective study that analyzed risk factors for PID identified three predictors for PIM: history of treated periodontitis, absence of regular supportive peri-implant maintenance, and use of a bone graft [60]. The same investigation also identified three predictors for PI: smoking, absence of regular supportive peri-implant maintenance and placement of ≥2 implants.

Standardization of PICF sampling is relevant due to the atypical morphology of the implant prosthesis. The insertion of different paper strips, cones, membranes or other devices would be technique sensitive and may offer misleading results. Biomarker concentration in the collected PICF may be adjusted for the collected volume due to the higher volume in inflamed tissues. This controversial confounder was rarely discussed in PICF studies.

Biomarkers are not necessarily present in a single moment of PICF collection due to several systemic or local factors. Most studies were cross-sectional which leads to another limitation. Due to a cyclic progression of peri-implant diseases, the immune-inflammatory event biomarkers responsible for tissue breakdown may not always be active in cross-sectional studies with a single moment of fluid collection [42]. Thus, studies on bone markers are often inconclusive despite bone loss being one of the main features of peri-implantitis. Very few longitudinal studies sampled implants over time. Some were interventional trials, sampling the diseased implants before and after therapy. Results showed that clinically stable treatment outcomes of peri-implantitis are associated with lower levels of putative pathogens total bacterial load and with reduction of IL-1β, IL-6, and VEGF levels in PICF [61]. Longitudinal studies may confirm the concentrations of biomarkers at specific sites and would theoretically show the shifts of such concentrations in diseased implants over time. These longitudinal investigations may aid in presenting biomarkers that predict the shift from health to disease or predicting the deterioration of a diseased implant. The validity of predicting PID is still in its infancy.

### 2.6. Future Directions: Genetics, Metabolomics, Prediction of Future Disease Progression

#### 2.6.1. Genotype and Polymorphism

Host susceptibility is a critical determinant in periodontal disease pathogenesis. There was little support in the literature for a specific genotype or phenotype of immune reactivity that could be reliably used as an indicator of susceptibility to peri-implant disease. While there are varying reports of associations of specific genotypes with peri-implantitis, the studies are inconsistent [62]. In certain population groups, there is some evidence that about twelve polymorphisms could be related to biological complications in implantology [63]. Some studies reported a positive association between polymorphism of IL-1 gene and PI. One study showed a negative association between polymorphism of TNFα and an increased risk of PI [64]. Another trial investigated genes associated with pathogenesis in PI and concluded that dysfunction of regulation in metal ion concentration might affect cell morphology or cell adhesion, resulting in implant failure [65]. Due to the scarcity of literature on this topic and some inconsistent results, more studies are necessary to further our knowledge in this topic.

#### 2.6.2. Metabolomics

The protein composition of GCF may reflect the pathophysiology of periodontal diseases. Protein profiles of GCF obtained from healthy individuals may potentially serve as a reference for identification of biomarkers of periodontal diseases by proteome analyses [20]. The same may be attempted for peri-implant disease and health. A recent report demonstrated that a specific PICF proteomic profile associates with the active peri-implantitis process and implant loss compared to the proteomic profile of healthy implants [66].

Metabolomic analysis (a comparative analysis of metabolome levels between samples) that measure small degradation molecules associated with host and bacterial metabolism show promise [20]. Metabolite functions include metabolism and energy storage, as well as other functions in cell-to-cell signaling, metal acquisition, and virulence [67,68]. Mass spectrometry and nuclear magnetic resonance spectroscopy can be used to both identify and quantify chemicals from complex mixtures such as PICF. This particular approach is now being exploited to characterize the metabolomes of many different biological samples in what is called quantitative metabolomics or targeted metabolic profiling [69]. Metabolomics offers unique insights into small molecule regulation and signaling in biology. Cancer-specific signatures have been shown to be embedded in saliva metabolites [70]. Identification of a molecular signature for periodontitis using unbiased metabolic profiling could allow identification of biomarkers to assist in the diagnosis and monitoring of periodontal disease [71]. Untargeted metabolomics is the relative quantitation of a broad range of metabolites, both known and unknown, in different samples [67,68]. This untargeted approach allows the discovery of unknown biomarkers without bias (the need to choose a certain substance beforehand) to correlate it with PI. These potential biomarkers may be investigated in longitudinal trials which can assist in the prediction of future disease progression.

#### 2.6.3. Limitations of the Review

It is important to mention that this review has some limitations. The outcomes from this review are limited by the heterogeneity between studies. The review only confirms evidence reported by other authors about biomarkers in diagnosing PID, but it does not give the necessary meta-analysis results because of the heterogeneity of included studies. This narrative review did not utilize the search and analytical methods of systematic reviews. Another methodological limitation is that the risk of bias of included studies was not defined.

## 3. Conclusions

Diagnosing peri-implant diseases using periodontal probing and radiographs may be inaccurate and only provides a historical record of past disease rather than current disease activity. Developing biomarker technologies may offer possibilities in diagnostic application. Although more research is needed, the assessment of proinflammatory cytokines (IL-1β, TNFα, MMP-8) in the PICF may be of value to diagnose peri-implantitis and peri-implant mucositis but are, at this time, inappropriate to predict peri-implantitis because of the limited evidence of controlled longitudinal clinical trials. Commercially available chair-side diagnostic tests for MMP-8 to detect peri-implant diseases are promising. In addition to these biomarker tools already under study, future investigations could test a multi-biomarker approach, which has been shown to demonstrate higher validity than a single biomarker. Other promising directions include the use of untargeted metabolomics and interventional, longitudinal trials to identify a unique set of biomarkers in PICF. These trials may also be able to determine the biomarkers’ validity to diagnose peri-implantitis and predict which patients and which dental implants may be at risk of disease progression. As studies show the increasing prevalence of peri-implant diseases, increasing diagnostic and predictive accuracy may have a significant impact on dental care.

## Figures and Tables

**Table 1:** Keywords and abbreviations used in this narrative review.

PI	Peri-Implantitis
PIM	peri-implant mucositis
PID	peri-implant disease
GCF	gingival crevicular fluid
PICF	peri-implant crevicular fluid
MMP	matrix metalloproteinase
IL-1β	interleukin 1 beta
IL-6	interleukin-6
IL-1ra	interleukin-1 receptor antagonist
TNFα	tumor necrosis factor alpha
GM-CSF	granulocyte-macrophage colony-stimulating factor
MIP-1α	macrophage inflammatory protein-1α
RANK	receptor activator of nuclear factor kappa-Β
RANKL	receptor activator of nuclear factor kappa-Β ligand
sRANKL	soluble Receptor activator of nuclear factor kappa-Β ligand
OPG	osteoprotegerin
TIMP	tissue inhibitor of metalloproteinases
MPO	myeloperoxidase
tPA	tissue plasminogen activator
TRAP	tartrate-resistant acid phosphatase
CatK	cathepsin K
PAI	plasminogen activator inhibitor
VEGF	vascular endothelial growth factor
ICTP	C-telopeptide pyridinoline cross linkage of type I collagen
ELISA	enzyme-linked immunosorbent assay
AUC	area under the curve (receiver operating characteristic)

**Table 2:** Summary of commonly investigated peri-implant crevicular fluid (PICF) biomarkers in peri-implantitis.

Biomarker	Summary	References
**Cytokines:**		
TNFα	higher levels in diseased vs. healthy implants	Zani 2016 [33], Duarte 2009 [45], Faot 2015 [31], Ghassib 2019 [34]
IL-1β	higher levels in diseased vs. healthy implants	Gurlek 2017 [23], Hall 2015 [37], Casado 2013 [36], Ramseier 2016 [52], Wang 2016 [25], Schierano 2008 [35], Ghassib 2019 [34]
IL-10	negative correlation with diseased implants	Zani 2016 [33], Casado 2013 [36]
**Bone Markers:**		
sRANKL, RANK, OPG	mixed results or no correlation between biomarker and PID	Arikan 2008 [39], Arikan 2011 [42], Monov 2006 [41], Rakic 2013 [43]
Osteocalcin	mixed results or no correlation between biomarker and PID	Dursun 2016 [40], Cakal 2018 [44]
**Enzymes:**		
MMP-8	higher levels in diseased vs. healthy implants	Arakawa 2012 [53], Basegmez 2012 [50], Ramseier 2016 [52], Ma 2000 [51], Wohlfahrt 2014 [32], Ghassib 2019 [34], Sorsa 2011 [49]
MMP-9	higher levels in diseased vs. healthy implants	Ma 2003 [54]
MMP-13	higher levels in diseased vs. healthy implants	Ma 2000 [51]
Myeloperoxidase	higher levels in diseased vs. healthy implants	Dursun 2016 [40]
Elastase	higher levels in diseased vs. healthy implants	Dursun 2016 [40]
Cathepsin K (CatK)	higher levels in diseased vs. healthy implants	Hall 2015 [37], Dursun 2016 [40], Strbac 2006 [47], Yamalik 2012 [48]
